# The effect of tranexamic acid in patients with TBI: a systematic review and meta-analysis of randomized controlled trials

**DOI:** 10.1186/s41016-020-00196-z

**Published:** 2020-06-09

**Authors:** Chao-nan Du, Bo-xue Liu, Qing-fang Ma, Ming-fei Yang

**Affiliations:** 1 Graduate School, Qinghai University, Xining, Qinghai China; 2 Department of Neurosurgery, Xuzhou Central Hospital, Xuzhou, Jiangsu China; 3 Department of Neurosurgery, Qinghai Provincial People’s Hospital, Xining, Qinghai China

**Keywords:** Traumatic brain injury, Tranexamic acid, Mortality, Disability

## Abstract

To conduct a systematic review and meta-analysis and evaluate the effect of tranexamic acid in patients with traumatic brain injury. PubMed, EMBASE, and CENTRAL (Cochrane Central Register of Controlled Trials) were searched to identify randomized controlled trials and evaluate the effect of tranexamic acid in traumatic brain injury patients. The primary outcome was mortality. Two reviewers extracted the data independently. The random effect meta-analysis was used to estimate the aggregate effect size of 95% confidence intervals. Six randomized controlled trials investigating tranexamic acid versus placebo and 30073 patients were included. Compared with placebo, tranexamic acid decreased the mortality (RR = 0.92; 95% CI, 0.87–0.96; *p* < 0.001) and growth of hemorrhagic mass (RR = 0.78; 95% CI, 0.61–0.99; *p* = 0.04). However, tranexamic acid could not decrease disability or independent, neurosurgery, vascular embolism, and stroke. Current evidence suggested that compared with placebo, tranexamic acid could reduce mortality and growth of hemorrhagic mass. This finding indicated that patients with traumatic brain injury should be treated with tranexamic acid.

## Background

Every year, it was estimated that more than 60 million individuals suffer from traumatic brain injury (TBI) all over the world for a variety of reasons [1]. TBI was a serious health problem and one of the main causes of death and disability worldwide. Study indicated that intracranial hemorrhage was a common complication of TBI, which increased the risk of death and disability [2]. The formation of microplots and vascular occlusion was common after brain injury [3]. Tranexamic acid (TXA) can inhibit fibrinolysis by competing with lysine residues on the surface of fibrin, thus stabilizing clots and blocking the interaction between plasmin and plasmin. Because of the potential role of TXA in reducing the size of hematoma and preventing secondary brain injury, TXA is considered as a possible treatment to improve the clinical outcome of TBI. However, the effects of TXA in patients with TBI remain controversial. So far, a randomized controlled trial (RCT), the CRASH-2 trial [4], reported that TXA could reduce the mortality, especially within 3 h in TBI patients. Indeed, another RCT CRASH-3 trial [5] further proved the above conclusions. In order to provide the latest and most convincing evidence, we systematically reviewed the existing literature to study whether TXA could reduce the mortality of patients with TBI. The secondary objective was to evaluate the effects of TXA on disability or independent, vascular embolism (including myocardial infarction, deep vein thrombosis, and pulmonary embolism), and stroke in TBI patients.

## Literature search and selection criteria

The meta-analysis was conducted in accordance with the Cochrane Handbook for Systematic Reviews of Interventions [6] and reported in accordance with the PRISMA (preferred reporting item for system review and meta-analysis) statement [7]. PubMed, EMBASE, and CENTRAL (Cochrane Central Register of Controlled Trials) were searched through January 3, 2020, with no restrictions. The following search terms were used “traumatic brain injury”, “Glasgow Coma Scale”, “Glasgow Outcome Scale”, “craniocerebral trauma”, “acute brain injury”, and “tranexamic acid”. Two independent investigators (CD and BL) conducted a preliminary search, deleted duplicate records, screened the relevance of titles and summaries, and identified articles requiring further evaluation. We reviewed the full-text articles to assess eligibility. Disagreements were resolved by discussion with another investigator.

The inclusion criteria were as follows: (1) population: patients with TBI; (2) intervention: TXA (1 g in 100 ml of normal saline); (3) comparison: placebo; (4) outcome: the primary outcome was mortality, the second outcomes included disability or independent, growth of hemorrhagic mass, neurosurgery, vascular embolism (including myocardial infarction, deep vein thrombosis, and pulmonary embolism) and stroke; and (5) design: RCTs.

## Data extraction and quality assessment

Two reviewers (CD and BL) extracted data independently. The data were extracted from each study as follow: first author, year of publication, country, intervention characteristics (number of patients, age, intervention methods), comparison characteristics (number of patients, age, comparison methods), and data on primary and secondary outcomes. When we found duplicate reports of the same trial, we retained only the most complete study. Disagreements were solved by discussion with another reviewer. The Cochrane risk of bias tool was adopted by two independent reviews to assess the risk of bias for each RCT [8].

## Statistical analysis

Differences were expressed as relative risk (RR) with 95% confidence interval (CI). Meta-analyses were performed using a random-effects model accounting for heterogeneity. The statistical heterogeneity of different trials was evaluated by I^2^ statistic [9]. Study with I^2^ values over 50% was considered to have high heterogeneity [9]. For the main outcome of mortality, subgroup analysis was conducted according to the characteristics of patients (Glasgow Coma Scale [GCS]3-8 and GCS 9-15). *p* < 0.05 was considered statistically significant. All statistical analyses were performed using Revman 5.3 (Nordic Cochrane Center).

## Study selection and characteristics

**Fig. 1:**
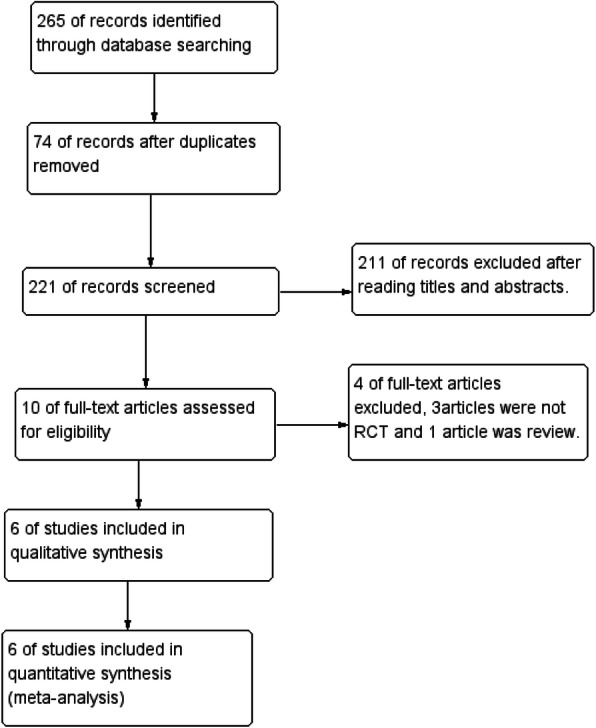
Study selection process

A total of 265 records were identified from the initial database search. Seventy-four records were excluded from duplicate records, and 211 were excluded for a variety of reasons, due to titles and abstracts (comments, letters, or not related to analysis). The remaining 10 full-text articles were assessed eligible, and four were excluded. Finally, 6 studies [4, 5, 10–13] were included in this meta-analysis. The selection process is shown in Fig. 1. The main characteristics of the included studies are shown in Table 1. These included studies were published from 2011 to 2019, and the sample sizes were 30073 (TXA group, 15089; placebo group, 14984). All studies were published in English.

**Table 1:** Characteristics of the included studies

	Trail	Country	Tranexamic acid group				Placebo group		
			Number	Age (years)	Method	Follow-up	Number	Age (years)	Follow-up
1	Chakroun-Walha 2019 [11]	Tunisia	96	44.0 ± 20.0	VTA was administered as soon as possible, with a first dose of 1 g in 100 ml of normal saline in 10 min and then with a maintenance dose of 1 g per 500 ml of normal saline for 8 h	28 days	84	39 ± 18	28 days
2	CRASH-2 trial collaborators 2011 [10]	India and Colombia	123	36.0 ± 14.0	VTA was administered with the first dose of 1 g in 100 ml of normal saline in 10 min and then with a maintenance dose of 1 g per 1000 ml of normal saline for 8 h	28 days	126	37 ± 14	28 days
3	CRASH-3 trial collaborators 2019 [5]	UK	4613	41.7 ± 19.0	VTA was administered with the first dose of 1 g in 100 ml of normal saline over 10 min, followed by an intravenous infusion of 1 g over 8 h	28 days	4514	41.9 ± 19.0	28 days
4	Fakharian 2018 [12]	Iran	74	42.3 ± 18.3	VTA was administered with the first dose of 1 g in 100 ml of normal saline in 10 min and then with a maintenance dose of 1 g per 1000 ml of normal saline for 8 h	3 months	75	39.3 ± 18.1	3 months
5	Roberts 2013 [4]	UK	10060	34.6 ± 14.1	VTA (loading dose of 1 g of VTA infused over 10 min, followed by an intravenous infusion of 1 g over 8 h)	28 days	10,067	34.5 ± 14.4	28 days
6	Yutthakasemsunt 2013 [13]	Thailand	120	34.8 ± 16.0	VTA (loading dose of 1 g over 30 min followed by a maintenance dose of 1.0 g infused over 8 h)	24–32 h	118	34.1 ± 15.3	24–32 h

**Fig. 2:**
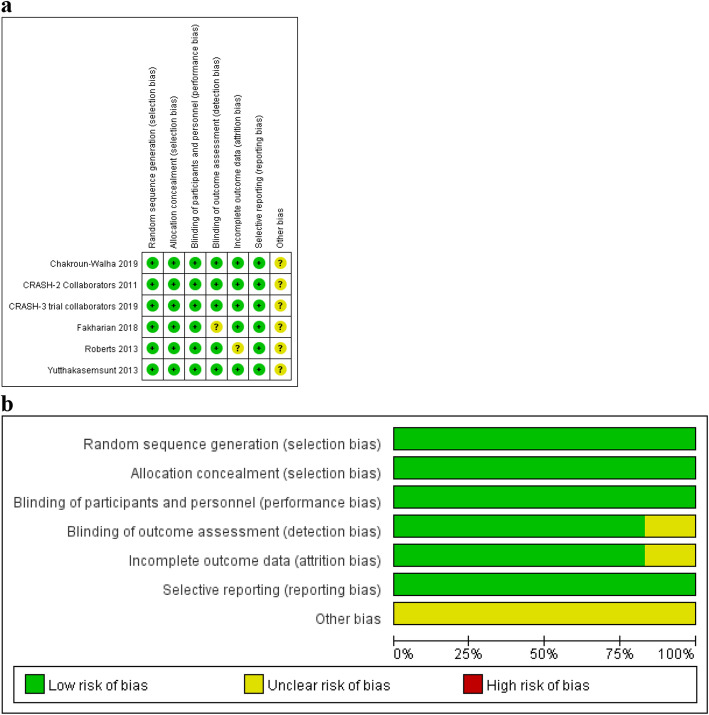
Risk of bias graph and bias summary. **a** + = low risk, and ? = uncertain risk.

All included studies reported mortality [4, 5, 10–13], three studies reported growth of hemorrhagic mass [10, 12, 13], four reported disability or dependent [4, 5, 12, 13], three studies reported neurosurgery [11–13], three reported vascular embolism [4, 5, 11], and three reported stroke [4, 5, 13]. Details of the risk of bias graph (Fig. 2a) and risk of bias summary (Fig. 2b) were presented. Overall, none of the study had high risk of bias (Table 2).

**Table 2:** Quality assessment of the included trials

Criteria	Design	Quality assessment				
		Concealment	Intention-to-treat analysis	Blinding	Outcome reporting bias	Quality of evidence
Chakroun-Walha 2019 [11]	Randomized trials	Yes	Yes	Yes	None detected	High
CRASH-2 trial collaborators 2011 [10]	Randomized trials	Yes	Yes	Yes	None detected	High
CRASH-3 trial collaborators 2019 [5]	Randomized trials	Yes	Yes	Yes	None detected	High
Fakharian 2018 [12]	Randomized trials	Yes	Yes	Yes	None detected	High
Roberts 2013 [2013]	Randomized trials	Yes	Yes	Yes	None detected	High
Yutthakasemsunt 2013 [13]	Randomized trials	Yes	Yes	Yes	None detected	High

## Primary outcome

**Fig. 3:**
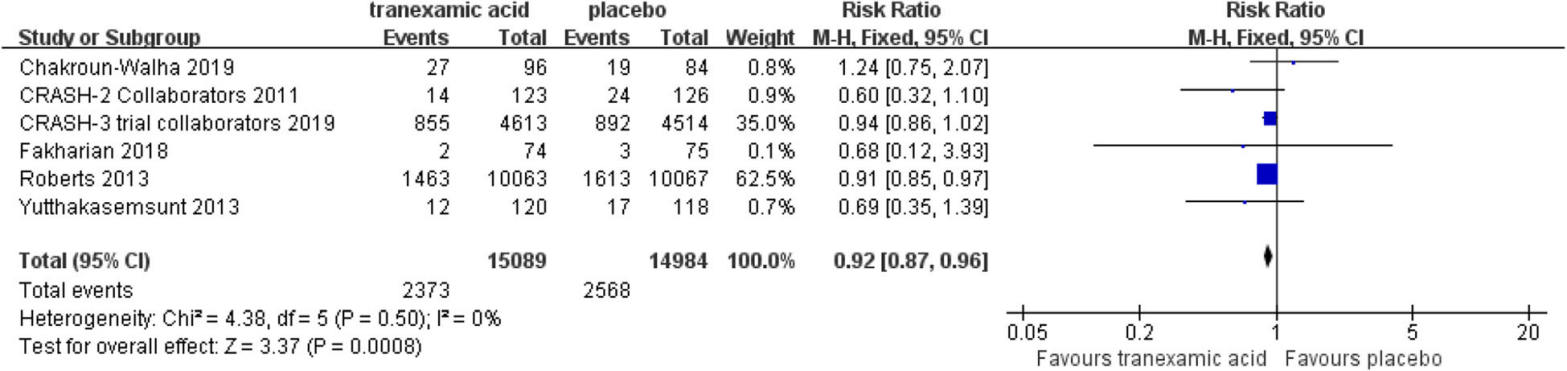
Forest plot of the meta-analysis of mortality. The results indicated that tranexamic acid could decrease the mortality significantly

**Fig. 4:**
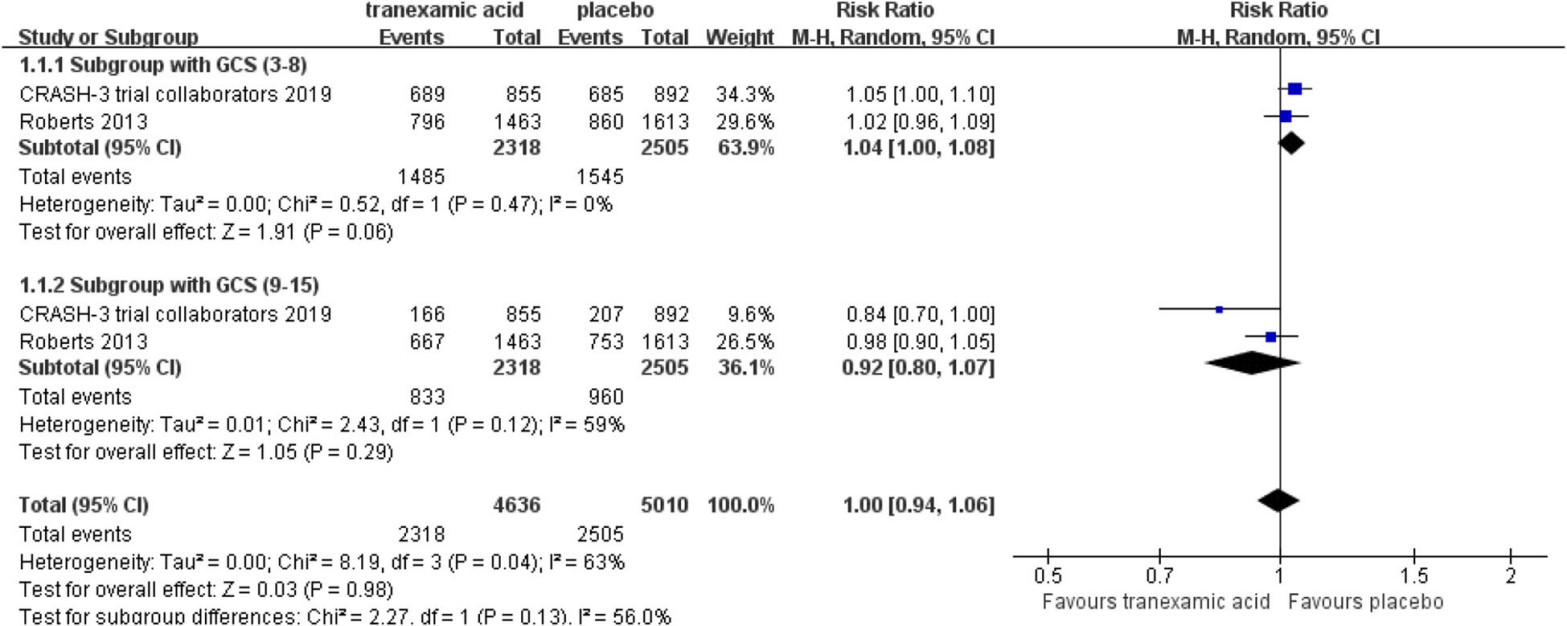
Forest plot for mortality according to the GCS 3-8 and GCS 9-15. The results indicated that there was no significance of patients with Glasgow coma scale 9–15 and Glasgow coma scale 3–8

The total number of mortalities was 4941 among the six trails. In TXA and placebo group, the mortality was respectively 15.7% (2373 of 15,089) and 17.1% (2568 of 14,984). TXA decreased the mortality significantly (RR = 0.92; 95% CI, 0.87–0.96; *p* < 0.001; Fig. 3). No statistical heterogeneity was observed in the trial (I^2^ = 0%). There was no significance of patients with GCS 9-15 (RR = 0.92; 95% CI, 0.80–1.07; *p* = 0.29) and GCS 3-8 (RR = 1.04; 95% CI, 1.00–1.08; *p* = 0.06; Fig. 4).

## Sensitivity analysis

The meta-analysis of mortality had no heterogeneity among the included studies, and thus we did not perform sensitivity analysis.

## Secondary outcomes

**Fig. 5:**
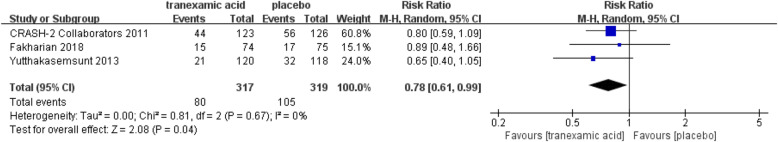
Forest plot of the meta-analysis of growth of hemorrhagic mass. The results indicated that there was significant in growth of hemorrhagic mass

**Fig. 6:**
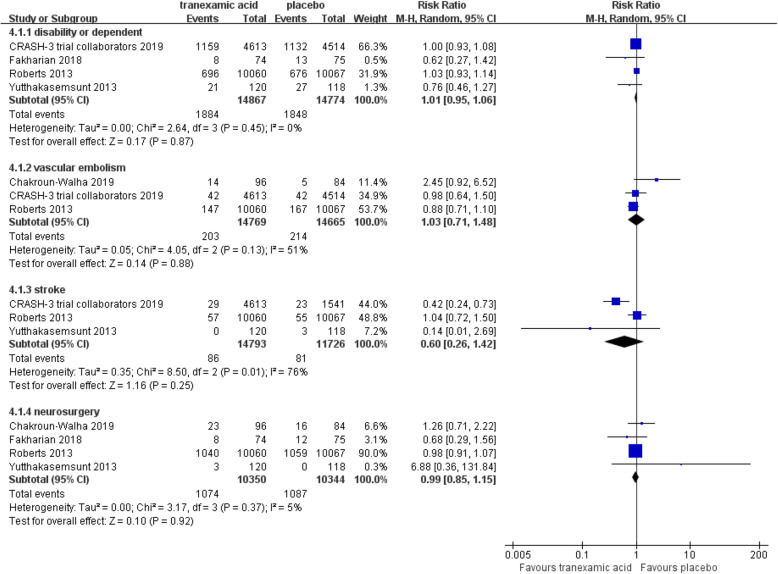
Forest plot of the meta-analysis of disability or independent, vascular embolism, stroke and neurosurgery. The results indicated that there were no significant differences in disability or independent, vascular embolism, stroke, and neurosurgery between tranexamic acid and placebo

There was significance in growth of hemorrhagic mass (RR = 0.78; 95% CI, 0.61–0.99; *p* = 0.04; Fig. 5). However, there were no significant differences in disability or independent (RR = 1.01; 95% CI, 0.95–1.07; *p* = 0.84; Fig. 6), vascular embolism (RR = 1.02; 95% CI, 0.70–1.48; *p* = 0.92; Fig. 6), stroke (RR = 1.07; 95% CI, 0.78–1.48; *p* = 0.67; Fig. 6), and neurosurgery (RR = 0.99; 95% CI, 0.85–1.15; *p* = 0.92; Fig. 6) between TXA and placebo.

## Main findings and comparison with previous studies

Compared with placebo, TXA (a first dose of 1 g in 100 ml of normal saline in 10 to 30 min after admission) could reduce the mortality and growth of hemorrhagic mass of patients with TBI. However, neither GCS 3-8 nor GCS 9-15 could reduce the risk. In addition, there was no significant difference between TXA and placebo in disability or independent, neurosurgery, vascular embolism, and stroke.

Several meta-analysis comparing TXA and placebo had been published [14–17]. Two meta-analysis of RCTs indicated that TXA had significantly reduced intracranial hemorrhage progression but not mortality [14, 17]. The other two meta-analysis further testified the progress of intracranial hemorrhage; however, the two meta-analysis demonstrated that TXA was associated with substantially reduced mortality [15, 16]. These above meta-analysis [14–17] with TBI, and proved that TXA could reduce the mortality. Moreover, subgroup analysis found that treatment with TXA between GCS 3-8 and GCS 9-15 did not reach significance.

Crash-2 trials [10, 18] showed that in TBI patients, TXA was given early administration (within 3 h after injury) significantly reduced mortality. In CRASH-3 trails [5], the safety of TXA in TBI patients had been confirmed and treatment within 3 h could decrease the mortality. Our data indicated that there was significance in growth of hemorrhagic mass (RR = 0.78; 95% CI, 0.61–0.99; *p* = 0.04). The current evidences showed that TXA had the effect of reducing bleeding and mortality. Study showed that Africa and Southeast Asia were the high incidence areas of TBI [1]. Two studies [4, 5] in this meta-analysis included the population of Africa and Southeast Asia, proved the effect of TXA, especially within 3 h after injuries. Therefore, TXA might be used in patients with TBI.

A large number of patients with TBI might had disability or dependent and attributed to heavy burden on the society. In this meta-analysis, we analyzed whether TXA could drop the disability or independent, but the results indicated that it could not. Meanwhile, TXA did not decrease the incidence of neurosurgery, vascular embolism, and stroke. Thus, more RCTs might be implemented to explore how to reduce the disability or dependent.

This meta-analysis supported the effect of decreasing the mortality and growth of hemorrhagic mass by comparing TXA with placebo. However, in this meta-analysis, long-term follow-up were not included in this study. More RCTs might be carried out in the future.

## Conclusion

Current systematic review and meta-analysis indicated that compared with placebo, TXA (a first dose of 1 g in 100 ml of normal saline in 10 to 30 min after admission) could reduce the mortality and growth of hemorrhagic mass in patients with TBI.

## Data Availability

Not applicable.

## Abbreviations

TBI: Traumatic brain injury
CENTRAL: Cochrane central register of controlled trials
TXA: Tranexamic acid
RCT: Randomized controlled trial
RR: Relative risk
CI: Confidence interval
GCS: Glasgow coma scale
